# Pharmacokinetics and lung delivery of PDDS-aerosolized amikacin (NKTR-061) in intubated and mechanically ventilated patients with nosocomial pneumonia

**DOI:** 10.1186/cc8206

**Published:** 2009-12-10

**Authors:** Charles-Edouard Luyt, Marc Clavel, Kalpalatha Guntupalli, Jay Johannigman, John I Kennedy, Christopher Wood, Kevin Corkery, Dennis Gribben, Jean Chastre

**Affiliations:** 1 Service de Réanimation Médicale, Groupe Hospitalier Pitié-Salpêtrière, Assistance Publique-Hôpitaux de Paris, Université Paris-Pierre-et-Marie-Curie, 47 boulevard de l'Hôpital, 75651 Paris Cedex 13, France; 2 Service de Réanimation Médicale, Centre Hospitalier Dupuytren, 2 avenue Martin Luther King, 87000 Limoges, France; 3 Critical Care, Baylor College of Medicine, One Baylor Plazza, Houston, TX 77030, USA; 4 Department of Surgery, Division of Trauma/Critical Care, University of Cincinnati, 2600 Clifton Avenue, Cincinnati, OH 45221, USA; 5 Division of Pulmonary and Critical Care Medicine, University of Alabama, 1802 6th Avenue South, Birmingham, AL 35249, USA; 6 Critical Care Department, University of Tennessee Health Science Center, 920 Madison Avenue, Memphis, TN 38163, USA; 7 Novartis Pharmaceutical Corp, 150 Industrial Road, San Carlos, CA 94070, USA; 8 Talima Therapeutics, 75 Shoreway Road, San Carlos, CA 94070, USA

## Abstract

**Introduction:**

Aminoglycosides aerosolization might achieve better diffusion into the alveolar compartment than intravenous use. The objective of this multicenter study was to evaluate aerosol-delivered amikacin penetration into the alveolar epithelial lining fluid (ELF) using a new vibrating mesh nebulizer (Pulmonary Drug Delivery System (PDDS), Nektar Therapeutics), which delivers high doses to the lungs.

**Methods:**

Nebulized amikacin (400 mg bid) was delivered to the lungs of 28 mechanically ventilated patients with Gram-negative VAP for 7-14 days, adjunctive to intravenous therapy. On treatment day 3, 30 minutes after completing aerosol delivery, all the patients underwent bronchoalveolar lavage in the infection-involved area and the ELF amikacin concentration was determined. The same day, urine and serum amikacin concentrations were determined at different time points.

**Results:**

Median (range) ELF amikacin and maximum serum amikacin concentrations were 976.1 (135.7-16127.6) and 0.9 (0.62-1.73) μg/mL, respectively. The median total amount of amikacin excreted in urine during the first and second 12-hour collection on day 3 were 19 (12.21-28) and 21.2 (14.1-29.98) μg, respectively. During the study period, daily through amikacin measurements were below the level of nephrotoxicity. Sixty-four unexpected adverse events were reported, among which 2 were deemed possibly due to nebulized amikacin: one episode of worsening renal failure, and one episode of bronchospasm.

**Conclusions:**

PDDS delivery of aerosolized amikacin achieved very high aminoglycoside concentrations in ELF from radiography-controlled infection-involved zones, while maintaining safe serum amikacin concentrations. The ELF concentrations always exceeded the amikacin minimum inhibitory concentrations for Gram-negative microorganisms usually responsible for these pneumonias. The clinical impact of amikacin delivery with this system remains to be determined.

**Trial Registration:**

**ClinicalTrials.gov Identifier: **NCT01021436.

## Introduction

Aminoglycosides are broad-spectrum antibiotics active against most Gram-negative pathogens responsible for ventilator-associated pneumonia (VAP), hospital-acquired pneumonia (HAP) or healthcare-associated pneumonia (HCAP), even those with multidrug-resistance patterns [1]. However, the systemic use of this antibiotic class is limited by its toxicity and poor penetration into the lung [2-4]. Also, minimum inhibitory concentrations (MIC) of still active antibiotics on multidrug-resistant Gram-negative bacteria, mainly aminoglycosides, are higher. Aerosol administration offers the theoretical advantage of achieving high antibiotic concentrations at the infection site and low systemic absorption, thereby avoiding renal toxicity [5]. Although available data are abundant for cystic fibrosis, data on aerosolized antibiotics for mechanically ventilated patients are scarce, even for aerosolized aminoglycosides, which are the most studied [6]. Moreover, during mechanical ventilation, high amounts of the particles dispersed by conventional nebulizers remain in the ventilatory circuits and the tracheobronchial tree before reaching the distal lung and, therefore, less drug is available in the alveolar compartment.

The Pulmonary Drug Delivery System (PDDS; Nektar Therapeutics, San Carlos, CA, USA) is a new vibrating mesh nebulizer designed to provide an estimated 40 to 50% of the dose administered to the lungs of intubated and mechanically ventilated patients, according to *in vitro *and *in vivo *data [7,8]. This high efficiency is explained by the device, which combines a high-performance generator and a breath-synchronized controller: the aerosol generator, which makes droplets 3 to 5 microns in diameter, consists of a proprietary high-frequency vibrating element that creates a rapid pumping of liquid droplets through tapered apertures to form the aerosol. The controller delivers aerosol only during the first 75% of each inspiratory phase. The combination of the two minimizes the impaction of aerosol droplets in the ventilatory circuits [9].

To evaluate amikacin penetration into the alveolar epithelial lining fluid (ELF), we performed a pharmacokinetic study on mechanically ventilated patients with Gram-negative nosocomial pneumonia receiving amikacin via the PDDS.

## Materials and methods

### Protocol and patients

The purpose of this multicenter (n = 6) trial was to evaluate the pharmacokinetics of PDDS-delivered aerosolized amikacin, combined with intravenous antibiotics, for patients with Gram-negative VAP, HAP or HCAP. Patients were included when they were aged 18 years or older, mechanically ventilated, had nosocomial pneumonia (defined as the presence of a new or progressive infiltrate(s) on chest radiograph and at least two of the following: fever, defined as core temperature >39.0°C or hypothermia, defined as core temperature <35.0°C; leukocyte count ≥10,000/mm^3 ^or ≤4,500/mm^3^; and new onset of purulent sputum production or respiratory secretions, or a change of sputum characteristics [10,11]); and a Gram-negative organism was detected by Gram-staining of tracheal aspirates. Non-inclusion criteria were: primary lung cancer or another malignancy metastasized to the lung, known or suspected active tuberculosis, cystic fibrosis, AIDS, or *Pneumocystis jiroveci *pneumonia; severe hypoxemia (partial pressure of oxygen/fraction of inspired oxygen (FiO_2_) ratio <100 mmHg); renal failure (serum creatinine >2 mg/dL or currently on dialysis); immunocompromised status; neutropenia; body mass index of 30 kg/m^2 ^or more; severe burns (>40% of total body surface area); refractory septic shock; known respiratory colonization with amikacin-resistant Gram-negative rods; and/or having received amikacin within the preceding seven days. After inclusion, patients received 400 mg of aerosolized amikacin twice daily (800 mg per day) for 7 to 14 days. Every patient's trough serum amikacin concentrations were measured daily. Patients who did not receive three full days of study medication were excluded.

For the study, a specially prepared, preservative-free formulation of amikacin sulfate formulated for inhalation (NKTR-061) was used for aerosolization, not a standard intravenous preparation. This solution contained amikacin sulfate at a concentration of 125 mg/mL; pH and osmolarity were adjusted for inhalation. Prior to starting studies in humans, inhalation toxicology studies were performed to make sure the dose was safe for inhalation.

The Institutional Review Board of each participating center approved the protocol, and informed consent was obtained from patients or their legally authorized representative prior to enrollment.

### Nebulizer

**Figure 1:**
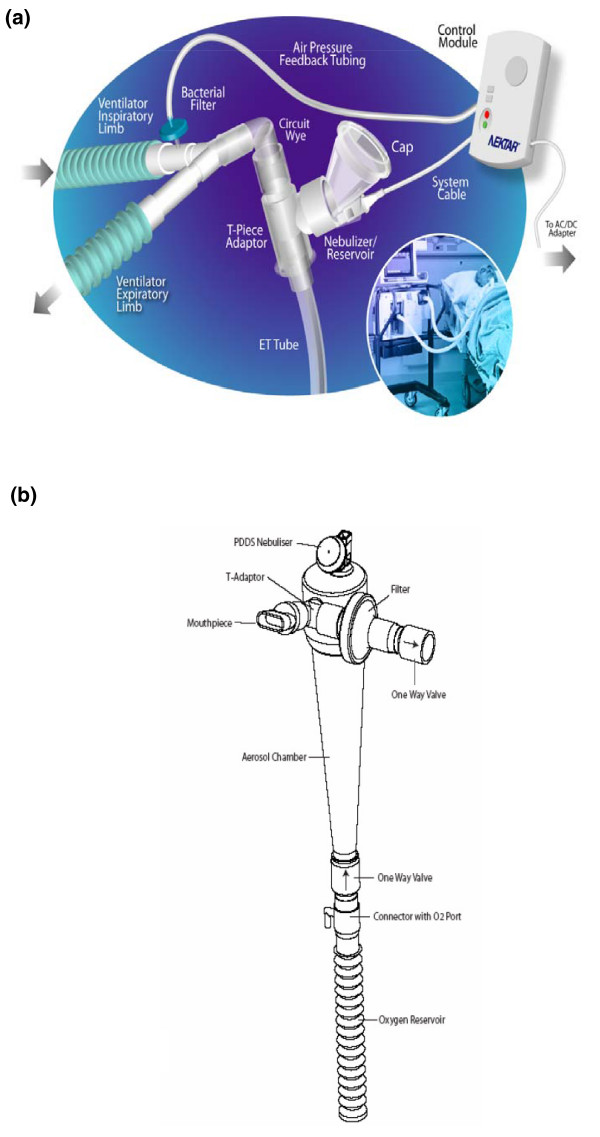
The pulmonary delivery drug system. **(a) **Clinical in the 'on-vent configuration' and **(b) **with the hand-held device.

The PDDS Clinical consists of a nebulizer/reservoir unit, T-piece adapter, air-pressure feedback unit for breath synchronization and a control module (Figure 1a). The nebulizer/reservoir unit, which is breath-synchronized and provides aerosol during the first 75% of inspiration, comprises the OnQ^® ^aerosol generator and a conical 6.25 mL drug reservoir, which contains the entire dose and requires no refilling. The aerosol generator consists of a proprietary high-frequency vibrating element that creates a rapid pumping of liquid droplets (of 3 to 5 μm) through tapered apertures to form the aerosol. The aerosol-generating process is electronically controlled via the control module. The nebulizer/reservoir unit is connected to the ventilator circuit through a T-piece adapter between the Wye-piece and the endotracheal tube. A cable connects the nebulizer/reservoir to the control module. The air pressure-feedback (for breath-synchronization) unit is connected to the inspiratory limb of the ventilator circuit and to the control module by pressure tubing.

The PDDS is a specialty drug delivery system for single-patient use. It is designed to deliver medication to adult patients on mechanical ventilation. The PDDS nebulizer/reservoir unit operates in phasic, breath-synchronized mode only, providing aerosol during the first 75% of inspiration during mechanical ventilation. This is accomplished by the control module sensing a positive pressure breath through the air pressure feedback tube. Limiting the aerosol formation to the first 75% of the inspiratory cycle enhances efficiency of delivery and minimizes exhaled aerosol. The duration of nebulization is dependent upon the patient's minute ventilation. The aerosol delivery time varies between 45 and 60 minutes [12]. The PDDS is an investigational device and is not commercially available.

### Nebulization technique

During nebulization, patients had to receive positive-pressure ventilation (i.e., pressure-control or volume-assist control modes). A heat-moisture exchanger or heated humidifier could be used with the device. For aerosolization, 3.2 mL of amikacin sulfate was added in the reservoir.

Aerosols were continued after extubation, the nebulizer/reservoir unit was attached to a reservoir unit with a mouthpiece, one-way valves and an expiratory filter (Figure 1b). In this configuration, the aerosol was generated continuously (not only during inspiration), and nebulization of the dose was completed in approximately 15 to 20 minutes in a previous study [12].

### Procedures

Fifteen to 30 minutes after the end of the first aerosolized dose given on day 3, all patients underwent fiberoptic bronchoscopy with bronchoalveolar lavage (BAL) in an infection-involved zone, as previously described [13]. After premedication with intravenous sedatives and a short-acting paralytic agent if needed (left to the discretion of the treating physician), the FiO_2 _was adjusted to 95% or more. The fiberoptic bronchoscope was advanced to the bronchial orifice selected on the basis of the radiographic infiltrate location. BAL was performed by instilling a total of at least 120 mL of sterile, non-bacteriostatic saline. The liquid recovered after the first aliquot was discarded, and the remaining BAL fluids were filtered through sterile gauze and pooled. The time between BAL onset and the total recovery of the six aliquots was kept as short as possible to minimize free diffusion of solutes, particularly urea, through the alveolar epithelium during the procedure. The entire procedure was well tolerated by all the patients. All efforts were made to keep the BAL specimen processing time as short as possible. BAL fluid samples were frozen and stored at -35°C until analyzed, i.e., determinations of ELF volume (V_ELF_) and amikacin concentration.

After starting the first day 3 aerosol, blood was drawn to measure serum amikacin concentrations at 30 minutes, and 1, 3, 6, 9, 12 and 24 hours, and cumulative urine samples, 0 to 12 and 12 to 24 hours, were collected to determine amikacin excretion via the kidneys. Serum creatinine levels were determined daily in each center's laboratory, according to local practices. Tracheal aspirates were collected on day 3 after the first aerosol and during the following 24 hours. Although tracheal suctioning was routinely performed by the nurses, tracheal aspirates collection was not compulsorily requested in the protocol and thus not performed in all patients: only 19 had tracheal aspirates collection for amikacin concentration determination. Moreover, because tracheal aspirates were collected as part of routine care, they were collected at different times for each patient. All samples were frozen and stored at -35°C until analyzed.

### Analytical measurements

The determination of amikacin concentrations in serum, tracheal aspirates and BAL, and urea levels in serum and BAL were performed by MEDTOX Laboratories (Saint Paul, MN, USA). All methods were pre-validated according to current Food and Drug Administration guidelines.

### Determination of V_ELF _recovered by BAL

As previously described [14,15], the V_ELF _was evaluated using urea as an endogenous marker of ELF dilution. Because urea diffuses easily and rapidly throughout the body, ELF and plasma urea concentrations are the same. In this setting, knowing the urea concentration in plasma and the urea quantity in a lavage sample enables V_ELF _to be calculated, as follows: V_ELF _= (BAL volume × (urea) in BAL)/(urea) in plasma, where (urea) is the urea concentration. Once the recovered V_ELF _is known, then any acellular component concentration (e.g., amikacin) can be calculated from it. The urea contents of BAL fluid samples were determined using a commercially available kit (Abbott Clinical Chemistry Urea Nitrogen Kit; Abbott Diagnostics, Abbott Park, IL, USA), and subsequently validated for analyzing urea in BAL. The urea content in corresponding serum samples was determined using the same kit without modification of the methodology as specified by the manufacturer.

### Determination of amikacin in serum

Serum samples drawn on day 3 were analyzed for amikacin over a concentration range of 200 to 500 ng/mL using an high performance liquid chromatography-mass spectrometry (HPLC-MS)/MS-based methodology. This methodology was used because commercial techniques for measuring amikacin were not sensitive enough to measure the expected serum levels in this study. Serum samples were mixed with internal standard (tobramycin) and 800 μL of 2% trichloroacetic acid and 200 μL of acetonitrile. Samples were then centrifuged and filtered through C18 extraction cartridges. The sample effluent was then analyzed using a 100 × 2.1 mm Betasil C18 column (Thermo Scientific, Waltham, MA, USA) and a mobile phase starting at 80% 1.5 mM heptafluorobutyric acid and 14% methanol and 6% water. The mobile phase was changed stepwise to a final composition of 80% methanol 20% water over the course of two minutes.

Amikacin was monitored using the specific fragmentation reactions produced under electronspray ionization - mass spectrometry (ESI-MS)/MS conditions on an ABI-Sciex 5000 triple quadrupole mass spectrometer (Applied Biosystem, Foster City, CA, USA). Amikacin was quantified by summing the transitions 586.2>425.2, 586.2>163.1 and 586.2>264.2.

Furthermore, trough serum amikacin concentrations before the morning nebulization were determined daily during the treatment period. Dosages were performed at each center using the kits available locally, with detection thresholds differing from one site to another. When the concentration was below the detection threshold, the latter was arbitrarily given as the value.

### Determination of amikacin in BAL

The BAL amikacin concentration was analyzed using a commercially available Syva^® ^Emit^® ^kit (Siemens Healthcare Diagnostics, Deerfield, IL, USA), designed for the analysis of amikacin in human serum. The methodology was modified to allow the analysis of amikacin in BAL over a concentration range of 2.50 to 50.00 μg/mL by simply preparing assay calibrators and quality-control samples in BAL fluid; no further modification of the assay procedure was required. This methodology was validated by performing an analytical method validation in full accordance to Food and Drug Administration guidelines and current bioanalytical industry practice.

### Pharmacokinetic analyses

The maximum serum amikacin concentration after the first dose on day 3 was defined as C_max_, with the time to C_max _defined as T_max_. The area under the serum amikacin concentration-time curve after the first dose (AUC_0-12 hour_) was calculated from the experimental data points obtained after the first dose on day 3 (30 minutes, and 1, 3, 6, 9 and 12 hours) using the trapezoidal method.

To determine amikacin absorption during the study period, amikacin concentrations were measured in the two day 3 urine collections, which reflected the quantity of each 12-hour dose absorbed via inhalation.

Because day 3 tracheal aspirates were not collected at specific time points, the 24-hour collection time was divided into four equivalent six-hour periods and then all results obtained during the corresponding period were pooled. The first period (H1 to H6) corresponds to the first six hours following the first day 3 aerosol, the second (H7 to H12) to the next six hours (before the second aerosol of the day), the third (H13 to H18) to the six hours following the second day 3 nebulization, and the fourth (H19 to H24) to the last six hours of the day, before the next aerosol.

All results are expressed as medians (interquartile range (IQR)), unless specified otherwise.

## Results

The characteristics of the 30 patients included in this study are reported in Table 1; 28 patients with VAP were included (no patients with HAP or HCAP were included) in the pharmacokinetic study after the specimens from two patients were excluded because these patients did not meet the requirement of receiving at least three full days of study medication to be included. All these 28 patients were on mechanical ventilation at day 3 (both nebulization of day 3), either through an endotracheal tube or a tracheotomy. Throughout the study, the median (IQR) duration of nebulization was 36 (30 to 45) minutes for intubated patients on mechanical ventilation. Median (IQR) duration of the 22 nebulizations for extubated patients using the handheld device was 20 (20 to 25) minutes.

**Table 1:** Characteristics of the 30 patients with Gram-negative VAP*

Parameter	Value
Age (year), median (IQR)	49 (33-57)
Sex, n (%)	
Male	23 (77)
Female	7 (23)
Body height at inclusion, cm, median (IQR)	177 (167.9-182.9)
Body weight at inclusion, kg, median (IQR)	84 (78-91)
Primary reason for MV, n (%)	
Trauma	13 (44)
Cardiac failure	4 (13)
Postoperative respiratory failure	4 (13)
Acute respiratory distress syndrome	3 (10)
Coma/CNS disease	3 (10)
Sepsis	2 (7)
Pulmonary embolism	1 (3)
Tracheotomy, n (%)	7 (23)
Septic shock at inclusion, n (%)	2 (7)
Vasopressor use at inclusion, n (%)	5 (17)
MV duration before VAP onset, days, median (IQR)	9 (5-11)
Acute respiratory distress syndrome at inclusion, n (%)	5 (17)
PaO_2_/FIO_2 _ratio upon inclusion, mmHg, median (IQR)	210 (171-281)

*Two of these patients were not included in the pharmacokinetic analysis because they did not received at least three full days of study medication.

**Figure 2:**
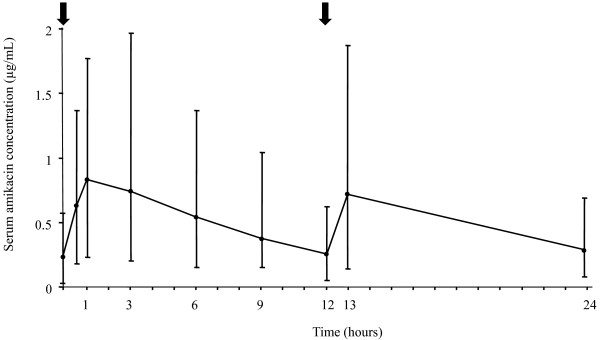
Day 3 serum amikacin concentrations before (0), and at hours 0.5, 1, 3, 6, 9, 12, 13 and 24 after starting the first aerosol. Results are expressed as medians (interquartile range). Black arrows indicate the timing of aerosols.

CNS = central nervous system; FiO2 = fraction of inspired oxygen; IQR = interquartile range; MV = mechanical ventilation; PaO2 = partial pressure of arterial oxygen; VAP = ventilator-associated pneumonia;

The median day 3 serum amikacin concentrations for the 28 patients are shown in Figure 2. Median (IQR) C_max _and T_max _were 0.85 (0.67 to 1.01) μg/mL and 1.0 (1 to 3) hours, respectively. AUC_0-12 hour _for amikacin was 6.15 (4.73 to 9.57) μg.hr/mL. The median total amount of amikacin excreted in urine during the first and second 12-hour specimens were 19 (12.21 to 28) and 21.2 (14.1 to 29.98) μg, respectively.

**Figure 3:**
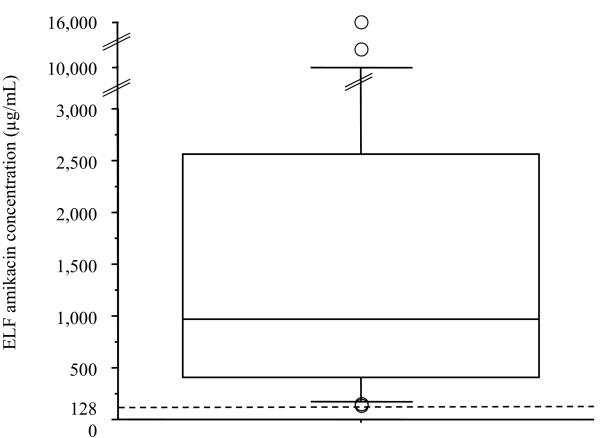
Day 3 amikacin concentration in the alveolar epithelial lining fluid (ELF) of the 28 assessable patients. The dotted line corresponds to 128 μg/mL, which is 10-fold the critical 90% minimum inhibitory concentration (MIC_90_) for *Pseudomonas aeruginosa*. T-bars represent the 10th and 90th percentiles; the horizontal line in the box is the median; the lower and upper limits of the box represent the 25th and 75th percentiles, respectively. Circles represent outliers.

**Figure 4:**
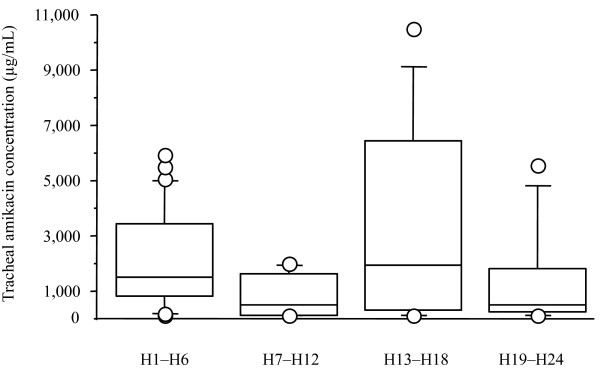
Day 3 amikacin concentration in the tracheal aspirates of the 19 assessable patients. H1 to H6 corresponds to the first six hours following the first aerosol, H7 to H12 to the next six hours (before the second aerosol of the day), H13 to H18 to the six hours following the second nebulization, and H19 to H24 to the last six hours of the day, before next aerosol. T-bars represent the 10th and 90th percentiles; the horizontal line in the box is the median; the lower and upper limits of the box represent the 25th and 75th percentiles, respectively. Circles represent outliers.

Fifteen to 30 minutes after the end of nebulization on day 3, the median amikacin concentration in ELF was 976.07 (410.33 to 2563.12) μg/mL, with respective lower and upper values of 135.67 and 16,127.56 μg/mL (Figure 3). Median V_ELF _was 0.46 (0.27 to 0.86) mL. No correlations could be established between the ELF amikacin concentration and ventilator settings (respiratory rate, peak inspiratory flow rate, mode of ventilation), presence of acute respiratory distress syndrome at the time of inclusion or ventilation duration. Tracheal aspirates for amikacin concentration determinations were collected on day 3 from 19 patients at 69 time points (Figure 4). Median amikacin concentrations for the four six-hour periods (H1 to H6, H7 to H12, H13 to H18 and H19 to H24) were: 1517.5 (793 to 3430), 477 (100 to 1605.5), 1948 (288.25 to 6412.5) and 472 (241.5 to 1825.5) μg/mL, respectively.

**Figure 5:**
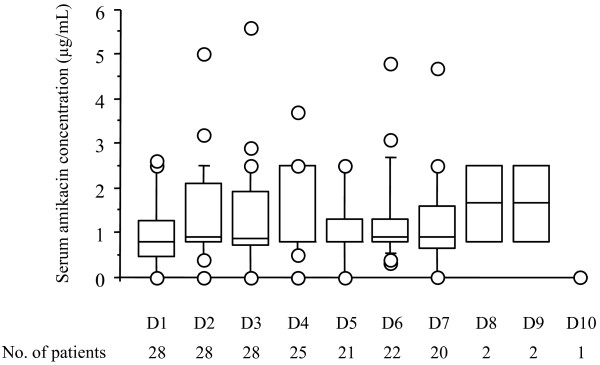
Serum amikacin trough concentrations during the study from day 1 (D1) to D10 with the corresponding number of patients. T-bars represent the 10th and 90th percentiles; the horizontal line in the box is the median; the lower and upper limits of the box represent the 25th and 75th percentiles, respectively. Circles represent outliers.

Patients were exposed to the study drug for a median of 7 (3 to 9) days. Figure 5 shows the trough serum amikacin concentrations during the study period. Values on day 1 (before any nebulization) were not null because the limits of detection varied from one center to another. Mean creatinine levels fluctuated between 53 and 106 μmol/L with no apparent trend.

Among the 64 unexpected adverse events reported in our study, one episode of worsening renal failure was possibly due to nebulized amikacin. The patient, who developed septic shock and was receiving many concomitant nephrotoxic medications, developed acute renal failure requiring continuous renal replacement therapy and aerosol discontinuation. The investigator deemed this severe adverse event possibly attributable to nebulized amikacin. Another patient experienced an episode of bronchospasm that resolved after discontinuing the amikacin and nebulizing bronchodilators.

## Discussion

In this study, we were able to demonstrate that amikacin, delivered by PDDS aerosolization, achieved high concentrations in the lower respiratory tract, in zones corresponding to radiographic infiltrate location, with low systemic absorption. Moreover, amikacin concentrations in ELF were more than 10-fold higher than the MIC_90 _of microorganisms usually responsible for nosocomial pneumonia (8 μg/mL for *P. aeruginosa*) [16]; and the observed amikacin concentrations exceeded the MIC_90 _of *Acinetobacter *species by four-fold [17]. Thus, based on this pharmacokinetic study, amikacin, nebulized via the PDDS, could have particular relevance for patients with Gram-negative VAP.

Aminoglycosides, combined with an antipseudomonal β-lactam, were recently proposed as an initial empiric antimicrobial regimen for patients with late-onset VAP or risk factors for multidrug-resistant pathogens [1]. But their lung penetration is poor [2]. The results of two studies showed that ELF penetration of gentamicin and tobramycin after intravenous infusion was poor, 12% and 32%, respectively, with peak concentrations below 10-fold the MIC of pathogens usually responsible for VAP [3,4].

Data on the bioavailability of aerosolized antibiotics in mechanically ventilated patients are scarce. Goldstein and colleagues found that amikacin nebulization, using an ultrasonic device, achieved high tissue concentrations in piglets, far above the MIC of most Gram-negative strains [5]. Those data were obtained in mechanically ventilated piglets with healthy lungs, but were confirmed in piglets with experimental *Escherichia coli *pneumonia: after nebulization, amikacin concentrations in lung tissue were 3 to 30-fold higher than after intravenous administration and were associated with a lower lung bacterial burden [18]. In humans, Le Conte and colleagues observed that a single tobramycin aerosolization delivered to patients with healthy lungs achieved high lung concentrations and low serum concentrations [19]. The same authors performed a multicenter, randomized, double-blind, placebo-controlled trial evaluating aerosolized tobramycin for patients with bacterial-proven VAP. They included 38 patients, among whom 21 received tobramycin and 17 a placebo, and showed that aerosols were well-tolerated. As all patients received, in addition to aerosols, intravenous tobramycin, the authors could draw no conclusions as to the efficacy or pharmacokinetics of the aerosol administration [20].

In an observational study conducted 10 years ago [21], Palmer and colleagues treated six patients, colonized with multidrug-resistant bacteria, with aerosolized gentamicin or amikacin. They showed that this antibiotic delivery route decreased the volume of tracheal secretions and bacterial burden in the tracheal aspirates. In their study, tracheal aminoglycoside concentrations were very high, without high systemic absorption in patients with normal renal function [21].

Only a few pharmacokinetic data are available on nebulization with vibrating mesh nebulizers. One study, conducted on six healthy volunteers receiving non-invasive pressure-support ventilation through a mouthpiece, used the Aeroneb^® ^Pro with a spacer. Amikacin was nebulized (40, 50 and 60 mg/kg). The authors showed that nebulizing up to 60 mg/kg of amikacin was safe and well-tolerated, with absorption estimated at 10 to 13% of the nebulizer load. However, those data were obtained in healthy volunteers and with non-invasive ventilation [22]. Two studies compared drug delivery with a vibrating mesh versus an ultrasonic nebulizer: delivering either tobramycin *in vitro *[23] or ceftazidime in an animal model [8]. Neither study found any difference in the amount of drug delivered, regardless of the type of nebulizer used. However, the Aeroneb^® ^Pro nebulizer, which is not breath-synchronized, was used in those studies and it can be thought that the amount of drug delivered to the lung would probably be higher using a breath-synchronized device [9].

Our findings are in accordance with a preliminary study, performed within the framework of a double-blind, placebo-controlled study of PDDS-delivered aerosolized amikacin in ventilated patients with Gram-negative VAP [24]. In that study, eight patients receiving aerosolized amikacin underwent two BAL: one in an infection-involved zone and the other in a radiologically normal zone. All patients had high amikacin concentrations in the tracheal tree, but also in ELF, even in poorly aerated zones [24].

One of the key problems with using aminoglycosides is their toxicity. In animals with healthy lungs, daily amikacin nebulization was not associated with tissue or systemic accumulation [25]. The same results were obtained in humans with healthy or infected lungs [19-21]. Our results showed that, despite high antibiotic levels in ELF and little systemic absorption, trough serum amikacin concentrations remained below the renal toxicity threshold [26]. Nevertheless, one patient experienced an episode of worsening acute renal failure that the investigator considered possibly related to the study medication.

The 400 mg dose was chosen based on a previous double-blind, placebo-controlled study of PDDS delivery of aerosolized amikacin to ventilated patients with Gram-negative VAP [27]. That study compared three regimens of two daily aerosolizations administered for 7 to 14 days: two regimens of nebulized amikacin (400 mg twice daily or 400 mg once daily and placebo), and placebo nebulized twice daily. The results showed that the 400 mg dose once or twice daily was sufficient to obtain high amikacin concentrations in tracheal aspirates (>25 μg/mL, the reference MIC for hospital-acquired organisms) with low trough serum concentrations, even in patients receiving amikacin twice daily, thereby avoiding renal toxicity [27]. In that study, patients given 400 mg of amikacin twice daily received less systemic antibiotics than patients receiving 400 mg once daily or placebo [12]. Moreover, a subgroup analysis showed that day 3 amikacin concentrations in alveolar ELF were very high [24].

Our study has several limitations. First, because all patients had normal renal function (a prerequisite for inclusion in the study), we cannot extrapolate our conclusions to patients with renal insufficiency or failure, which is frequent in intensive care patients with VAP. Although the diffusion into ELF might be the same, it is likely that the blood concentration would have been higher. Second, using urea as a marker of dilution could have underestimated the real concentration. Indeed, urea can leak into the air spaces during the BAL procedure, leading to overestimation of its concentration in BAL fluids and hence V_ELF_. Overestimating V_ELF _would have led to underestimation of amikacin concentrations in ELF. On the other hand, because of possible bronchial backflow during BAL collection, BAL fluids might have been contaminated by tracheal secretions, whose amikacin concentrations are very high, and that would have overestimated the concentrations [27]. Finally, amikacin concentrations varied widely among patients. This variability is probably due to multiple factors, including aeration, ventilator settings, ventilatory circuit and patient's specific factors. These factors may deserve to be evaluated in a specifically designed study. However, variability due to poor nebulization reproducibility cannot be excluded. But, the ELF amikacin concentrations always exceeded the MIC of microorganisms responsible for VAP; hence, these variations probably have no clinical implications.

## Conclusions

Amikacin aerosolization with the PDDS vibrating mesh nebulizer achieved high concentrations in ELF with little systemic absorption and accumulation, thereby confirming recent data obtained in healthy volunteers [22]. The clinical efficacy of adjunctive aerosol therapy remains to be determined.

## Key messages

• Amikacin aerosolization with the PDDS achieved high concentration in the trachea and alveolar epithelial lining fluid.

• Amikacin systemic absorption is low with this device.

• The clinical implication of nebulization with this device remains to be determined.

## Abbreviations

AUC: area under curve; BAL: bronchoalveolar lavage; Cmax: maximum serum amikacin concentration; ELF: epithelial lining fluid; FiO2: fraction of inspired oxygen; HAP: hospital-acquired pneumonia; HCAP: healthcare-associated pneumonia; IQR: interquartile range; MIC: miminum inhibitory concentration; PDDS: pulmonary delivery drug system; Tmax: time to maximum serum amikacin concentration; VAP: ventilator-associated pneumonia; VELF: volume of alveolar epithelial lining fluid.

## Competing interests

JC received lecture fees from Nektar Therapeutics. KC and DG were Nektar Therapeutics employees at the time of the study. The other authors declare that they have no competing interests.

## Authors' contributions

CEL, KC, DG and JC participated in the conception and design of the study, analyzed and interpreted the data, and drafted the manuscript. CEL, MC, KG, JJ, JK, CW and JC participated in data collection. All authors read and approved the final manuscript.
